# Clinical Features of Parkinson’s Disease: The Evolution of Critical Symptoms

**DOI:** 10.3390/biology9050103

**Published:** 2020-05-19

**Authors:** Csaba Váradi

**Affiliations:** Institute of Chemistry, Faculty of Materials Science and Engineering, University of Miskolc, 3515 Miskolc, Hungary; kemcsv@uni-miskolc.hu; Tel.: +36-30-8947730

**Keywords:** Parkinson’s disease, neurodegeneration, Lewy body, oxidative stress, clinical criteria

## Abstract

Parkinson’s disease (PD) is a multi-attribute neurodegenerative disorder combining motor and nonmotor symptoms without well-defined diagnostic clinical markers. The presence of primary motor features (bradykinesia, rest tremor, rigidity and loss of postural reflexes) are the most characteristic signs of PD that are also utilized to identify patients in current clinical practice. The successful implementation of levodopa treatment revealed that nonmotor features are the main contributors of patient disability in PD, and their occurrence might be earlier than motor symptoms during disease progression. Targeted detection of prodromal PD symptoms can open up new possibilities in the identification of PD patients and provide potential patient populations for developing novel neuroprotective therapies. In this review, the evolution of critical features in PD diagnosis is described with special attention to nonmotor symptoms and their possible detection.

## 1. Introduction

The discovery of Parkinson’s disease (PD) originated from James Parkinson, who described this disorder as “paralysis agitans’’ in 1817 [1]. Since then, PD has been defined as a complex neurodegenerative disorder expressing various symptoms due to the dopaminergic neuronal cell death in the substantia nigra (SN) [2]. PD is identified as the second-most common neuropathological disorder after Alzheimer’s disease associated with significant disability and decreased quality of life [3]. The incidence of PD is more frequent in industrialized countries and was found to increase with aging [4]. PD is very rare in people younger than 40 years of age, while 1%–2% of the world population is affected over 65 and 4%–5% over 85 [5]. Men-related higher incidence is described due to the protective effect of estrogen in women [6], but smokers [7] and coffee-consumers [8] are also facing lower risks, as nicotine is monoamine-oxidase-B (MAO-B), while caffeine adenosine A2a is a receptor antagonist, resulting in potential neuroprotective effects. In each year, 60,000 people are diagnosed with PD in the United States, causing a significant socioeconomic cost of ~20 billion dollars per year [9]. The main portion of this cost is the inpatient care at late disease stages, which is reportedly higher than disease medication; thus, there is an urgent need for early disease identification [10]. Due to the lack of accurate diagnostic tests, PD is characterized by the presence of motor and nonmotor symptoms using clinical classification criteria [11]. Most symptoms are progressive, and the monitoring of these cardinal features are the key decision markers of disease identification in the current clinical practice [12]. A clear indication of the diagnosis uncertainty is that the main confirmation of PD identification is the positive response to dopaminergic therapy [13]. One of the main problems in PD diagnosis is that every patient is different regarding their symptoms, severity and progression; thus, each person has their own version of PD, while cardinal features such as bradykinesia and rigidity can originate from normal aging or complex medical conditions as well [14]. The most common symptoms can also overlap with other neurodegenerative diseases, such as multiple system atrophy (MSA), progressive supranuclear palsy (PSP) and dementia with Lewy bodies (DLB) [15]. Another difficulty is that current therapies are only symptomatic, and even when the disease is identified, there are no available strategies to halt the neurodegeneration process. In this review, the critical features of PD and their use in clinical diagnostics are described, with special attention to the early symptoms and their detection.

## 2. Pathology

There is limited knowledge available on the onset of PD, although several features are associated with the development of the disease. Potential environmental risk factors include toxins (paraquat and rotenone) [16] and methanol exposure [17], carbon monoxide poisoning [18] and head trauma [19], while genetic mutations (SNCA, the gene of α-synuclein, LRRK2, the gene of leucine-rich repeat kinase 2 and GBA, the gene of glucocerebrosidase) [20] can also lead to neurodegeneration. These factors, along with aging, can induce mitochondrial dysfunction and increased oxidative stress, resulting in neuronal energy failure and neurodegeneration [21]. PD is also defined as a synucleopathy due to the abnormal accumulation of α-synuclein and subsequent intracellular aggregation leading to Lewy body (LB) formation [22]. α-synuclein, known as a presynaptic nerve terminal protein encoded by the SNCA gene, modulating synaptic vesicle recycling and neurotransmitter release [23]. It is mainly localized in the mitochondria of the neuronal cells, especially in the olfactory bulb, hippocampus, striatum and thalamus [24]. Mutations of the SNCA gene can reportedly influence the initiation of intracellular aggregates [25]. Although α-synuclein is the main component of LBs, they are actually composed of more than 90 different molecules, including not only PD-related gene products (SNCA and LRRK2) but also mitochondria-, ubiquitin-proteasome- and autophagy-related proteins [26]. Mutations on the encoding region of mitochondria are found to be expressed in impaired α-ketoglutarate dehydrogenase activity [27] and complex I defects [28], increasing mitochondrial dysfunction and oxidative stress. Altered proteolysis is identified as a potential molecular feature inducing neurodegeneration due to ubiquitin-proteasome and lysosome dysfunction [29]. Decreased heat shock protein 70 [30] and lysosome-associated membrane protein type 2A expression [31] are also found in PD, suggesting the altered proteasomal and lysosomal activity. Gaucher disease known as a lysosomal storage disorder is identified to provide 20–30-fold higher risk of developing PD due to the mutations in the gene of beta-glucocerebrosidase (GBA), suggesting the critical role of proteolysis in the initiation of neurodegeneration [32].

## 3. Cardinal Motor Symptoms in PD Identification

The presence of bradykinesia, rest tremor, rigidity and loss of postural reflexes are the most commonly identified motor symptoms of PD, although other clinical features can also be identified during disease progression, such as bulbar dysfunction, neuro-ophthalmological abnormalities and respiratory disturbances. One of the main signs that can improve the differentiation of PD from other parkinsonian disorders—thus, disease identification—is that most motor symptoms are responsive to dopaminergic therapy.

### 3.1. Bradykinesia

Bradykinesia is the most characteristic primary motor symptom of PD, defined by slow movement, decrementing amplitude and problematic fine motor control due to the decreased neuronal density in the SN. Patients with bradykinesia cannot provide sufficient energy to the executive muscles thus, fail to implement fast movements. Initial manifestations involve the slowness of reaction times and difficulties with performing simultaneous tasks, although impaired swallowing, loss of gesturing and decreased blinking can also be included [33]. Bradykinesia can be influenced by the emotional state of PD patients, requiring higher external triggers to access motor programs. The degree of dopamine deficiency is usually in good correlation with bradykinesia [34].

### 3.2. Tremor

One of the most easily recognized features of PD is the rest tremor, which is a rhythmic muscle contraction and relaxation mainly on the extremities but can expand to lips, chin and jaw. The characteristics of rest tremors in the hands is defined by supination and pronation, which can be decreased by action or during sleep. In patients with PD, a postural tremor is also described, which is an outstretched horizontal position against gravity. Postural tremor is found to be more prominent than rest tremor and might be one of the first signs of PD [35]. Both types of tremors are presented in the same (4–6 Hz) frequency range and responsive to dopaminergic therapy in contrast to essential tremor. In essential tremor, the head and voice can be affected with relatively higher frequency (5–10) and with positive response to alcohol, beta-blockers and botulinum toxin. Early-age essential tremor is reportedly a potential risk factor in developing PD [36].

### 3.3. Rigidity

Rigidity is the second-most common primary motor symptom of PD, which is described as the inflexibility of the limbs and neck or trunk. While in bradykinesia, the speed of the movement is reduced, in rigidity, the motion is limited to a reduced range due to muscle stiffness and the lack of relaxation capability. Typical symptoms of PD originating from rigidity are the painful shoulders, which is often misdiagnosed as arthritis [37]. Dopaminergic agonists are reportedly efficient in reducing rigidity.

### 3.4. Postural Instability

Postural instability is the main symptom of the lost postural reflexes, and usually manifests at late disease stages. This is the main responsible symptom for most falls of PD patients and subsequent hip fractures [38]. Postural instability is not responsive to dopaminergic therapy, although deep brain stimulation has been found to be promising [39].

## 4. Recognition of Nonmotor Features in PD

**Figure 1:**
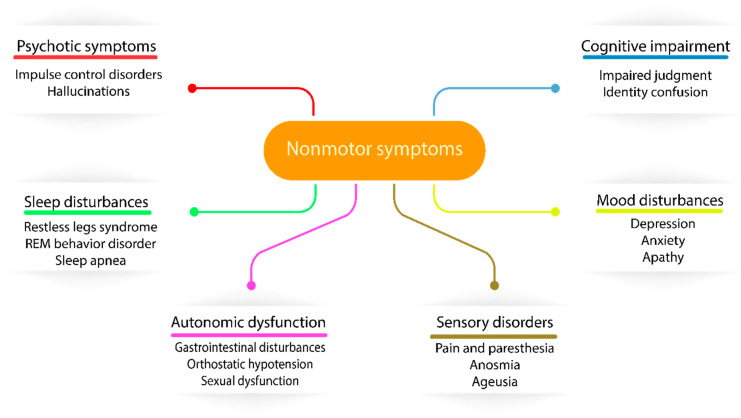
The diverse nature of nonmotor symptoms affecting Parkinson’s disease (PD) patients.

The first milestone in PD treatment originated from Carlson and coworkers, who identified PD-like symptoms in reserpine-treated rabbits due to dopamine uptake inhibition, suggesting the central problem of this disease [40]. As dopamine is impermeable through the blood-brain barrier, its precursor, called levodopa, was investigated in PD treatment, although the peripheral accumulation of dopamine was expressed in major side effects, such as nausea and vomiting [41]. This issue was solved by the combination of levodopa with a peripheral acting dopamine carboxylase inhibitor (carbidopa), enabling levodopa to enter the brain before degradation [42]. Since the introduction of dopaminergic therapy, long-term levodopa treatment was associated with fluctuations in motor responses and involuntary movements called dyskinesias [43]. The molecular explanation of this side effect is that striatal dopamine receptors are normally continuously activated due to the stable level of dopamine, although in levodopa-treated PD patients, the dopamine presence is more intermittent, as levodopa’s half-life is 60–90 min. The introduction of MAO inhibitors (preserve dopamine) and catechol-O-methyl-transferase (COMT) inhibitors (preserve levodopa) in PD treatment resulted in increased brain availability of levodopa and also reduced levodopa-induced dyskinesias due to the more controlled level of available dopamine [44]. The combination of these inhibitors (MAO and COMT) with carbidopa and levodopa became the most superior medication strategy in the latest years in PD therapy [45]. The improved motor features in response to levodopa resulted in the recognition of nonmotor symptoms of PD and suggested a change in the widely accepted cause of PD, namely the dopaminergic neuronal loss in the SN due to cytoplasmic α-synuclein aggregation and Lewy body production. In recent years, it has been identified that Lewy body neurodegeneration can affect the peripheral and enteric nervous systems through not only dopaminergic involvement but, also, glutamatergic, GABAergic, noradrenergic, serotonergic, histaminergic and cholinergic nerves. The most vulnerable nerves are usually poorly myelinated and have long-thin axons [46], including serotoninergic neurons in median raphe, cholinergic neurons of the nucleus basalis, epinephrine neurons of the locus coeruleus of Meynert, neurons in the olfactory system, cerebral cortex, spinal cord and peripheral autonomic nervous system [47]. The dysfunctionality of these nerves are expressed in an extremely wide range of nonmotor symptoms (NMS), such as sensory abnormalities, autonomic dysfunction, sleep disturbances and cognitive impairment neuropsychiatric symptoms (Figure 1), suggesting that PD is a complex disorder expressing both motor and nonmotor symptoms throughout the progression of the disease. Generally, all PD patients are affected by some of the NMS, with an increased frequency throughout the disease progression. NMS are one of the main problematic components of disease identification and treatment in PD due to their poor clinical diagnosis in current practice [48]. In terms of clinical diagnosis, NMS have crucial importance, as Lewy body pathology and α-synuclein deposition is found to be originating from the olfactory bulb and lower brain stem, spreading to the midbrain and cortical regions [49] and potentially appearing earlier than motor symptoms during the progression of the disease. The recognition of the spreading pathology of PD can also explain some of the fails of animal models where substantia nigra was directly targeted, but the expressed symptoms have not been matched to the overall PD symptoms [50].

## 5. The Importance of Prodromal Symptoms

**Figure 2:**
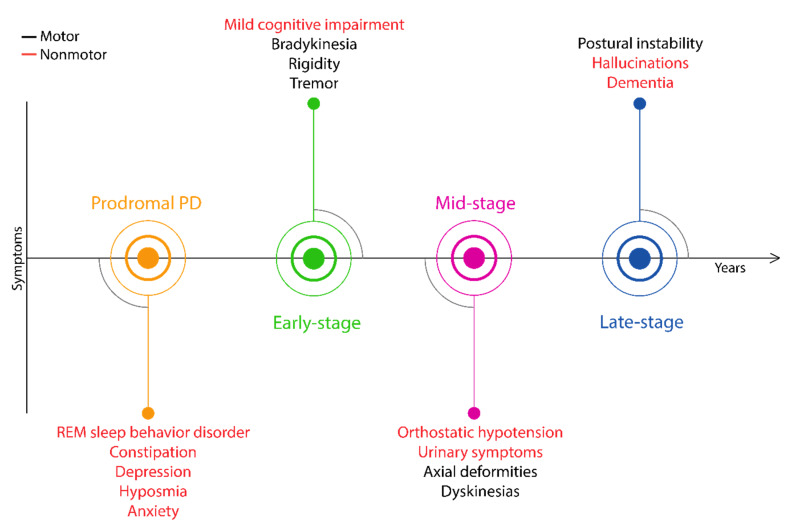
Timeline of clinical signs expressed throughout PD.

The proposed PD symptoms timeline suggests that the clinical onset of primary motor symptoms potentially occur years later than the disease has actually initiated [51]. The spreading pathology of PD advise that it is a multisystem disorder affecting the peripheral and central nervous systems, manifesting motor symptoms significantly later than the neurodegeneration has initiated [49]. In terms of the appearance of symptoms, PD can be separated into preclinical, prodromal and clinical stages [52]. In the first, preclinical stage, a contstant neurodegeneration is started in the substantia nigra, with no clinical signs. Then, it is followed by a >10-year prodromal stage with continuous neuronal loss but also expressing some nonmotor symptoms, as is visualized in Figure 2. After this stage, when 40%–60% of dopaminergic cells are lost, the first motor symptoms (bradykinesia, rigidity and tremors) manifest, and the patients enter the early stage of PD [53]. The lack of early markers results in constant neurodegeneration over a decade, until the prescence of motor symptoms and the possibility of disease identification. The earlier appearance of some nonmotor symptoms in prodromal PD increased their attention in the diagnostic procedure, including rapid eye movement sleep behavior disorder (RBD), constipation, olfactory loss and depression. The recognition of prodromal PD by the detection of early nonmotor symptoms could potentially initiate the development of novel neuroprotective therapies to intervene into PD progression. This could be key to counteracting the expression of more severe nonmotor and motor symptoms.

### 5.1. Rapid Eye Movement Sleep Behavior Disorder

Sleep disturbances are one of the main nonmotor symptoms in PD, including RBD, restless leg syndrome and sleep apnea. High percentages of people with sleep disorders are likely to develop PD, although RBD is found to be in the best correlation with PD progression. RBD is a type of parasomnia linked with repetitive dream enactment behavior and rapid eye movement (REM) sleep with the loss of atonia. It is often described as sleepwalking, although it is important to differentiate, as sleepwalking typically occurs in non-REM sleep, while there is rarely dreaming. RBD is identified by polysomnography, with subsequent muscle activity on the electromyogram channels. It has been associated with several types of synucleopathies, including multiple system atrophies, dementia with Lewy bodies and PD [54]. The estimated risk is significantly higher for developing PD in patients with RBD compared to the general population, and once PD is initiated, the motor progression is faster than in patients with no RBD. Compared to other early symptoms, RBD has the highest specificity and predictive value in disease identification, although the need for a polysomnography hinders its clinical use due to the necessitated time and expenses.

### 5.2. Constipation

Constipation is the result of less-frequent and/or more difficult bowel movements, one of the most common and disabling prodromal symptoms of PD, affecting more than 60% of PD patients [55]. It can originate from multiple causes, such as impairment of the autonomic nervous system, physical weakness, reduced fluid intake and medication side-effects (trihexyphenidyl), although it is associated with α-synuclein deposition as well [56]. Urinary dysfunction is usually considered as a potential risk factor in PD, although it has to be noted that it can also originate from chronic constipation. Clear advantages of constipation over RBD is that a single-question screening test is sufficient to analyze the general population.

### 5.3. Olfactory Dysfunction

Reduced odor sensitivity linked with PD has been described in 1975 by Ansari and Johnson [57]. Since then, olfactory dysfunction is widely accepted as an early symptom of PD [58]. More than 80% of PD patients are affected by hyposmia, suggesting a sensitive prodromal marker of the disease [59]. Olfactory receptor neurons are expressing odorant receptors and transmitting the detected odors as an electric signal to the nervous system through long unmyelinated axons, which is their potential weakness against neurodegeneration. According to the Braak staging, in stage 1, α-synuclein inclusion and corresponding Lewy body pathology initiate in the olfactory bulb, suggesting that olfactory loss is not just a prodromal symptom of PD but a potential first marker of the neurodegeneration preceding motor symptoms by years [60]. This could be a key point in PD identification, as by the detection of olfactory loss, potential neuroprotective therapies could be applied to inhibit or slow neurodegeneration. A major advantage of olfactory loss over other prodromal symptoms is that several olfactory testing kits are available without the need of an expert nurse or neurologist [61,62]. Olfactory dysfunction has several advantages over other prodromal symptoms in PD detection, although it can be manifested in other neurodegenerative disorders as well, thus using this as an independent predictive marker of PD is problematic.

### 5.4. Depression

Depression is a mood disorder referring to an altered emotional state of the patients and affecting personal behavior. The altered balance of neurotransmitters in the brain such as serotonin, norepinephrine and dopamine is mainly associated with depression, causing sadness and loss of interest. One of the main nonmotor symptoms of PD is depression, which affects ~50% of PD patients with a progressive manner, further developing anxiety and panic attacks. Constant fatigue and apathy have also been identified as some of those preliminary symptoms that can lead to depression. There are several tools available for depression identification, such as the geriatric depression scale (GDS-15) [63] and Montgomery-Asberg depression rating scale (MADRS) [64], although all of them require an expert to implement the assessment [65].

## 6. The Evolution of Critical Symptoms Involved in Diagnostic Procedures of PD

PD is a progressive disorder with multiple symptoms manifesting throughout the years, although there is no definitive sign that could allow direct disease identification. This has resulted in the concept of producing guidelines in the last decades, mainly based on the presence or absence of certain symptoms and responses to therapy. The aims of the developed criteria were to provide a potential route through the set of complex symptoms in order to identify and classify affected patients, thus reducing their disabilities and improving their quality of life.

The first attempt to describe PD onset and progression was provided by Hoehn and Yahr in 1967 [66]. In their study, patients were examined over a two-year period recording 77 features, such as health history, associated diseases, neurological signs, disease progression and the level of disability. Interestingly, aging and gender had no significant effect on the incidence of PD. Tremor and rigidity were found to be the most characteristic initial symptoms, although delayed initiation and slowness of movement were also described. They concluded that a more precise description of the individual cardinal symptoms is required to be able to correlate with the disease progression and identify definite, probable and possible parkinsonism. They also described the degree of disability in PD patients correlating with the disease progression, resulting in five different stages that is called the Hoehn and Yahr scale. Each stage has a defined motor impairment and disability by which patients can be classified during disease progression. This original scale was later modified by the Movement Disorder Society (MDS), although it is still one of the most commonly used rating scales in PD progression [67]. Two years later, Schwab and England have presented the activities of daily living (ADL) scale aiming to assess impaired mobility of PD patients during their daily activities [68]. This scale provides a percentage on patient daily living, depending on the symptoms expressed by the disease.

In 1988, the United Kingdom Parkinson’s Disease Society Brain Bank (UKPDSBB) provided the first form of clinical diagnostic criteria for PD by Gibb et al. [69]. This guideline has differentiated three different levels of the classification process, including diagnostic, exclusion and supportive criteria, as described below.

UKPDSBB clinical diagnostic criteria for PD:

1. Diagnostic criteria: presence of bradykinesia and at least one of the following symptoms: muscular rigidity, 4–6 Hz rest tremor and postural instability (not caused by other disorders)
2. Exclusion criteria: history of repeated strokes or head injury, encephalitis, early severe autonomic involvement or dementia, Babinski sign, negative response to levodopa treatment and MPTP (1-methyl-4-phenyl-1,2,3,6-tetrahydropyridine) exposure
3. Supportive criteria (at least three required): unilateral onset, rest tremor, progressive course, persistent asymmetry, excellent response to dopaminergic therapy, levodopa-induced dyskinesia, positive levodopa response five years or more and clinical course of ten years or more

Some of the main advances of UKPDSBB guidelines were that bradykinesia was considered the main motor symptom, and the response to dopaminergic therapy has also been involved in the diagnostic criteria. The frequency of the tremors and the cause of postural instability has been defined to exclude other neurological disorders. Moreover, detailed exclusion criteria have been created, including the negative response to levodopa treatment. Several other criteria have been developed in the last 20 years, although UKPDSBB guidelines have been one of the most widely employed in clinical trials and practice. In 1999, Gelb and colleagues described an improved criteria aiming to distinguish PD from other related conditions [70]. Similarly to previous studies [71,72], the Gelb criteria differentiated three levels of PD: definite, probable and possible. The main deviation was that they limited the diagnosis of definite PD to neuropathologic confirmation and claimed that only probable and possible PD can be identified by classification criteria. In contrast to the UKPDSBB guidelines, where the unilateral onset was only a supportive criterion, in the Gelb criteria, asymmetric onset was a cardinal sign along with a rest tremor, bradykinesia and rigidity. Patients with probable PD have to express these typical features at least for three years without the development of any other atypical symptoms, and also, a positive response to dopaminergic therapy is required, according to Gelb. The criteria for possible PD is more flexible, allowing less typical symptoms manifested over a shorter period of time than three years without the need of a good response to levodopa treatment.

The successful implementation of a levodopa treatment and corresponding motor improvement resulted in the increased attention of nonmotor features expressed by PD patients but also highlighted the weaknesses of UKPDSBB and Gelb criteria. The importance of nonmotor features and their effects on quality of life suggested the generation of improved guidelines, including nonmotor features. To address this issue in 2009, Lees and colleagues published a modified version of the Queen Square Brain Bank (QSBB) clinical diagnostic criteria [73]. This guideline was mainly based on the UKPDSBB criteria, extended for the first time with nonmotor features, such as hyposmia and hallucinations, and used as supportive criteria in PD diagnosis. This has been a major improvement, as, in the Gelb criteria, hallucinations were considered as a support for alternative diagnosis.

In 2015, the International Parkinson and Movement Disorder Society (MDS) task force reported a novel clinical diagnostic criteria designed for PD research and clinical practice called MDS-PD [74]. The UKPDSBB and Gelb criteria were the main basis of the MDS-PD development, while several nonmotor symptoms were considered as additional diagnostic features. Sleep, autonomic, psychiatric and olfactory dysfunction were all considered as common nonmotor features of PD, and their absences were used as red flags in MDS-PD, suggesting an alternative Parkinson-mimicking condition. This was a crucial milestone, as all the significant prodromal symptoms (RBD, constipation, olfactory dysfunction and depression) were included in the MDS-PD criteria.

## 7. Potential Future Perspectives

Although the developed guidelines are well-defined and detailed, one of their main limitations is the need of expert neurologists. The patient population is relatively older, and they usually carry multiple diseases, which can make more difficult the use of strict clinical diagnostic criteria. For accurate disease identification, more data is required that can be specified for PD. These can be supported by the analysis of molecular alterations or the use of smart devices detecting prodromal symptoms of PD.

### 7.1. Molecular Markers for PD Identification

One of the main future perspectives is the discovery of molecular alterations that can improve the identification of PD patients. Comprehensive meta-analysis of microarray datasets is a widely accepted strategy to provide information on the altered gene expression levels in pathological conditions. Using this technique, with the combination of the feature selection procedure and classification model, Jiang and colleagues have found 1229 genes with altered expressions (640 upregulated and 589 downregulated) in PD patients compared to healthy controls. Nine of the identified alterations were mainly related to the circadian cycle, sleep and gonadotropin-releasing hormone signaling pathway and showed excellent diagnostic values, suggesting potential novel biomarkers of PD [75]. Sakharkar et al. applied a systems biology approach to identify gene expression alterations in PD comparing brain gene expression profiles and peripheral blood cells [76]. They identified 316 genes down- and 98 upregulated in the brain, and 20 of these were differentially expressed in the blood as well. The top three interacting genes with altered expressions were APP, EGFR and PARK2, suggesting the potential cause of dopaminergic neuronal cell death.

While a comprehensive meta-analysis of PD tissues and blood samples is reportedly helpful, targeting prodromal symptoms and associated molecular alterations might be more sufficient in early PD detection. For example, Wen et al. have recently reported an altered microstructural network of olfactory dysfunction in newly diagnosed PD patients, claiming that the disconnectivity of the bilateral olfactory circuitry could be a biomarker of the olfactory dysfunction in early PD [77]. To understand the background of intestinal lesions and constipation in PD patients, Li and colleagues analyzed the gut microbiota of patients with PD and compared to healthy controls. They found decreased diversity of species and altered relative abundance, especially of Akkermansia and Lactobacillus [78]. Wang et al. found decreased serotonin-1A receptor binding in patients with depression, suggesting serotonergic dysfunction in the hippocampus, raphe nuclei, insular, anterior cingulate cortex and occipital cortex [79], which has been described in PD patients as well [80]. Degeneration of GABA (γ-aminobutyric acid)-ergic and glycinergic neurons is also reported in RBD as a potential cause of lost atonia [81]. All these molecular alterations are providing novel possibilities in the early identification of PD patients, although the need of instrumentation and experts are preventing their routine clinical use in PD diagnostics.

### 7.2. Smart Devices

Another future perspective for early PD detection is the implementation of smart devices and wearable sensors for the identification of prodromal symptoms. Today, smart devices are widely accepted tools, mainly monitoring daily activity, sleep and exercise. The most commonly used smart devices that are related to human health are bands, watches and scales, although recently, several investigations have been reported addressing more critical health issues.

One of them is sleeping, which can influence our daily activity, mood and the overall quality of life; thus, home sleep monitoring has crucial importance in the identification of chronic disorders of sleep. In 2017, Tal and colleagues have reported a contact-free sleep monitoring system that is able to detect sleep stages, heart rate and respiratory rate in good correlation with polysomnography [82]. Considering the fact that polysomnography is the main tool to identify RBD, this could have huge potentials not only in the identification of sleep disorders but in the recognition of prodromal PD.

The concept of a smart toilet has been recently published by Park et al. aiming to detect infections, colon cancer and constipation by analyzing stools and detecting tumor DNA or even viral RNA [83]. As constipation is one of the prodromal symptoms of PD, smart toilets could potentially improve the early identification of PD patients, especially if the technology could be extended by the detection of α-synuclein in the stool, which is reportedly accumulated in the bowel of PD patients [84].

Depression is one of the most difficult to measure of the prodromal symptoms and usually necessitates lengthy clinical interviews and questionnaires. To overcome this issue, McGinnis and co-workers have developed a novel strategy to identify depression by the combination of wearable sensors and machine learning. They have used a 90-s fear-induction task by a commercially available wearable sensor and analyzed the generated data by machine learning to predict the diagnosis of anxiety and depression in young children. This is also a potential technology that could improve the earlier identification of PD patients by the detection of depression [85].

## 8. Conclusions

The lack of definite clinical markers for PD diagnosis has initiated the development of symptom-based guidelines for the identification and classification of PD patients. The most widely accepted clinical criteria are solely built on motor symptoms, although the successful implementation of dopaminergic therapy has pointed out the importance of nonmotor features. The patients’ quality of life and their degree of disability is highly affected by the presence of nonmotor symptoms, and more importantly, their appearance can precede motor symptoms by several years. The first diagnostic criteria for PD have not considered NMS as significant features or used as a suggestion of alternative criteria, while in current guidelines, their presence is essential in PD diagnosis. The involvement of several NMS into novel guidelines have improved diagnostic accuracy and sensitivity, although early-stage identification is still challenging. One of the potential strategies is to identify molecular alterations that can be specified for the prodromal symptoms of PD. The use of smart devices and wearable sensors can also provide essential data to identify prodromal symptoms of PD by analyzing sleep (RBD), defecation (constipation) and mental health (depression). These could help to recognize earlier the disease initiation and develop novel neuroprotective therapies to improve PD remission.
